# Stem Cell Research Funding Policies and Dynamic Innovation: A Survey of Open Access and Commercialization Requirements

**DOI:** 10.1007/s12015-014-9504-5

**Published:** 2014-03-28

**Authors:** Maroussia Lévesque, Jihyun Rosel Kim, Rosario Isasi, Bartha Maria Knoppers, Aurora Plomer, Yann Joly

**Affiliations:** 1 Centre of Genomics and Policy, McGill University, 740 Avenue du Docteur-Penfield, Montreal, Quebec Canada H3A 1A5; 2 School of Law, Bartolome House, The University of Sheffield, Winter Street, Sheffield, S3 7ND UK

**Keywords:** Stem cell research (SCR), Human embryonic stem cells (hESC), Induced pluripotent stem cells (iPSC), Open access, Data sharing, Commercialization

## Abstract

This article compares and contrasts the pressures of both open access data sharing and commercialization policies in the context of publicly funded embryonic stem cell research (SCR). First, normative guidelines of international SCR organizations were examined. We then examined SCR funding guidelines and the project evaluation criteria of major funding organizations in the EU, the United Kingdom (UK), Spain, Canada and the United States. Our survey of policies revealed subtle pressures to commercialize research that include: increased funding availability for commercialization opportunities, assistance for obtaining intellectual property rights (IPRs) and legislation mandating commercialization. In lieu of open access models, funders are increasingly opting for limited sharing models or “protected commons” models that make the research available to researchers within the same region or those receiving the same funding. Meanwhile, there still is need for funding agencies to clarify and standardize terms such as “non-profit organizations” and “for-profit research,” as more universities are pursuing for-profit or commercial opportunities.

## Introduction

Researchers have noted a potential conflict between open access and commercialization requirements in the field of biomedical research. Indeed, a recent overview of the data access policies of the principal science funding bodies in the United Kingdom reveals differences concerning monitoring and financial support for the implementation of open access policies [1]. Likewise, a summary review of the Canadian, British and American literature suggests a possible conflict between open access and commercialization in genomic academic research [2]. Using bibliometrics to visualize the effect of patenting on collaborative research in Canada as measured in co-authorship, another study reveals that commercial activity could have a negative impact on research collaboration [3]. There is also growing evidence that commercialization pressures are increasing and becoming more institutionalized through funding policies [4].

This article analyzes how commercialization and open pressures are represented in public funding documents and stem cell innovation frameworks in international, national and regional contexts. How complex are the current knowledge technology transfer requirements? Are there any visible contradictions between the various policies? Are there other policy issues that could create innovation bottlenecks? To explore these questions, we briefly introduce the international policy framework governing embryonic SCR, before turning to an analysis of specific funding bodies and organizations.

## Research and Methodology

We conducted a review of open access and commercialization policies—as established by publicly funded bodies—to investigate commercialization and open access pressures in the underlying innovation framework, and the level of policy integration between selected jurisdictions. While our study focuses specifically on hESC research, our analysis and conclusions are likely generalizable to other sources of pluripotent stem cell lines, such as iPSCs. We chose to focus in the EU, United Kingdom, Spain, Canada, and the United States because those jurisdictions met the following criteria: (1) high level of public (national/regional) funding, (2) leading science with regards to the stem cell field, (3) establishment of a national stem cell biobank and (4) documentation publicly available in English, Spanish and/or French.

Amongst our research questions were: are there clear proprietary or open science requirements embedded in funding policies and guidelines, including requirements for the translation of stem cell research? Are dissemination requirements or additional funding support for intellectual property rights (IPRs) used as pressures or incentives to promote one innovation approach over another? At the level of international organizations, countries, and specific regions, we studied the policy requirements of funding bodies, stem cell banks, and non-profit SCR research organizations as embodied in laws, guidelines, information documents and material transfer agreements (MTAs). Policies applicable to the translation of SCR into downstream research and clinical use were classified into two broad categories: open science requirements and intellectual property (patent) requirements.

Using the keywords “open access” and/or “commercialization” in the search engine of each surveyed organization, we examined SCR funding guidelines and project evaluation criteria. We performed additional searches on the websites of funding bodies using the following terms: access agreement, access license, commercialization, data sharing, intellectual property policy, intellectual property, IP, material deposit agreement, material transfer agreement, patent, research translation policy, research use license, research tool, royalty, and stem cell. Where the context warranted, we supplemented our general queries with institution-specific technical terms, such as ‘foreground IP’ for the FP7 program financed by the EU. Our search returned 94 documents.

Where available, we also used specialized briefs outlining the funding landscape for a particular jurisdiction. For the UK, we consulted the UK policy excerpts from the Hinxton Group (2006), the UK Stem Cell Initiative’s document “Estimated UK Investment in Stem Cell Research” [5] and a workshop summary on regenerative medicine [5]. For Canada, we referred to the Canadian Asset Map for Stem Cell and Regenerative Medicine [6]. For Spain, we consulted a paper published by Genome España [7]. For international sources, we relied on existing academic resources [8–10]. Finally, we also consulted centralized grant query portals [11] to confirm the thoroughness of our list of surveyed organizations. We confirmed the accuracy and completeness of the information reflected on organizations’ websites with the guidance of an expert team of advisors [12].

Our inclusion criteria were:

- International organizations. International organizations with a critical mass of researchers that seek to harmonize SCR practices and set global policy standards were included because of their possible normative influence on national SCR funding policies.
- International and national public funding organizations substantially promoting SCR activities. In order to determine the level of SCR activity, we considered the number of grant opportunities specifically applicable to SCR or with an explicit mention of SCR in a funding application, as well as the amount of funding. Our research focuses on public funding organizations because our hypothesis is that these organizations face heightened pressure to make their research open, whether by making their research results available for free (Wellcome Trust) [13] or publishing their results online in a peer-reviewed academic journal [14]. They also face increasing pressure to translate results to the clinic, for which commercialization via restrictive IP practices can be instrumental. SCR activity also includes stem cell banks that facilitate the procurement of SCR material.
- SCR regional and national policy and data results from 2010. Some jurisdictions, particularly the United States, lack SCR-specific access policies and legislations. Thus, we relied on the general laws and guidelines that impact the translation of SCR. Moreover, given the scarcity of material at the international level, we extended our search to 2006 in order to capture some key international documentation. We excluded most state or provincial laws and focused primarily on federal legislation, with the exception of California in the U.S. section to highlight its role as a forerunner in SCR [15, 16]. We also surveyed the autonomous community of Andalusia, as its legal framework, funding policies and institutional strategy provide a singular innovation model because of the adoption of the policy set forward by the Andalusian Initiative for Advance Therapy (AIAT). This flexible framework provides an interesting contrast to that applicable in California, the other region analyzed in our study.
- Data available in English, French or Spanish.

Our exclusion criteria were:

- Private, for-profit organizations. While many private funding bodies [17] have provided significant funding for SCR, they were excluded from our research because we wanted to examine how the public funding sector is affected by open access and commercialization pressures.
- Lack of public data available. Although some jurisdictions have high levels of SCR activity, the available literature does not allow an informed analysis of their innovation frameworks. We focused on the organizations located in the five jurisdictions with the most available information rich data (detailed policies, legal documents, and guidelines) for a clear picture of their national innovation framework.

### Definitions

In the context of this paper, we define commercialization as the process of extracting economic value from new products, processes, and knowledge through the use of IPRs, licensing agreements, and the creation of spin-off companies [18]. For the purpose of our research, rather than to enter the terminological debate that is often associated with open access movements and strategies, we chose to adopt a broad perspective on this notion to be as inclusive as possible [19]. Thus, we considered open access as the open sharing of scientific knowledge and research materials with minimum restrictions, with the international scientific community, to forge a model of open scientific collaboration. These models are intended to stimulate downstream investigations and analyses and to improve research translation [2].

## Results and Discussion

### International Organizations

International professional organizations such as the International Society for Stem Cell Research (ISSCR) and the Hinxton Group provide an institutional foundation for the development and dissemination of best practices [20]. These institutions have an important role in fostering scientific progress, policy development and influencing funding support. However, it is also important to acknowledge that their power is limited to that of “legitimacy pressures” [21]—in other words, proposing new, non-binding, policies and acting as a mediator between jurisdictions.

#### The International Society for Stem Cell Research (ISSCR)

The ISSCR is a non-profit organization for stem cell researchers. It fosters exchange and dissemination of information pertaining to stem cell research [22] and advocates open access.

The ISSCR Guidelines for the Conduct of Human Embryonic Stem Cell Research [23] were designed as an international ethical and professional guidance standard that could be incorporated in SCR grant requirements. The guidelines address all scientists, clinicians and institutions engaging in SCR, regardless of affiliation. ISSCR influences the ethics and policy recommendations of national organizations.

Their guidelines “endorse in the strongest possible terms the principle that research with human materials is valuable to all, and that the proper practice of science requires unhindered distribution of research materials to all qualified investigators engaged in non-commercial research and the dissemination of its benefits to humanity at large on just and reasonable terms” (s. 7.1) [23]. Further, “as a prerequisite for being granted the privilege of engaging in human stem cell research, researchers must agree to make the materials readily accessible to the biomedical research community for non-commercial research” (s. 7.2) [23]. The sample hESC-related MTA defines “commercial purposes” as “the sale, lease, license, or other transfer of the material or modifications to a for-profit organization” (s. I. 10) [24].

The ISSCR’s sample MTA also provides a template agreement for sharing SC, wherein the user must adhere to open access practices. This agreement is “not to be interpreted to prevent or delay publication of research findings” (s. II.11) [24], and recipients must preserve “reasonable public and scientific access” when distributing material derived from the sample (s. II.5(c)) [24]. They must also ensure the rights for the non-exclusive transfer of their material to academic and governmental research institutions for internal research purposes (s. II.5) [24]. In other words, they cannot use exclusive licenses to privatize downstream inventions building on open access material, without expressly written consent from the provider and after negotiating in good faith (s. II.6–7) [24].

In its Guidelines for the Clinical Translation of Stem Cells [25], the ISSCR encourages development and “assessment of alternative models of intellectual property, licensing, product development, and public funding to promote fair and broad access to stem cell-based diagnostics and therapies” (s. 38(c)(ii)) [25]. This invites researchers to consider novel IPR regimes that give wider access to therapeutic products arising from their SCR.

Most importantly, the ISSCR invites funding institutions and researchers to adopt the principles of open science. It explicitly endorses open access as a scientific obligation, provides a model MTA to facilitate material sharing and promotes wide access to both research tools and therapeutic products [25].

#### The Hinxton Group

Funded by non-profit organizations such as the Wellcome Trust and the Greenwall Foundation as well as governmental bodies (e.g. UK Foreign and Commonwealth Office), the Hinxton Group is an institution born from an “interdisciplinary group to explore the ethical and policy challenges of transnational scientific collaboration raised by variations in regulations governing embryo research and stem cell science” [26]. While its role is advisory [27], the Hinxton Group’s recommendations may influence the scope of public funding policies due to its advocacy role in promoting open access and encouraging funders to maintain the accessibility of fundamental research tools.

The Hinxton Group has highlighted the dangers associated with current SCR patenting practices, pointing out that “the tree-like shape of cellular differentiation makes the field especially prone to IPR holdings that can function as tollbooths to access broad areas of work, creating a drag on investment and slowing down basic research” [27]. It therefore proposes alternative IPR arrangements that would limit exclusivity in two ways. First, the Hinxton Group encourages patent owners to facilitate access to their material by non-profit institutions [27]. Second, it prompts researchers to prioritize data sharing by using patent pools or formally constructed semi-commons to prevent exclusive licensing and overcome the patent thicket issue [27]. This point is related to technologies needed to advance the stakes of the field [27], not downstream development of commercial (or therapeutic) applications.

The Hinxton Group also takes the position that cordoning off fundamental research like “novel cell lines, reagents and related technologies [that] function as platforms for broad areas of follow-on work” [27] is particularly deleterious to the advancement of SCR. It invites decision makers to compel SC researchers to implement open access measures and “develop a consistent policy for sharing data and materials post publication.” [27].

One weakness of the Hinxton Group’s statement is the lack of acknowledgement of the blurred lines between “non-profit” and “for-profit” institutions. This is particularly evident in their recommendation pertaining to the licensing of government-funded SCR inventions, which state that they must “reserve research rights for non-profit institutions” and “[e]nsure that data and materials are available to government and academic researchers with a minimum of delay” [27]. From their statement, it remains unclear whether “non-profit institutions” exclude universities when they pursue partnerships with private companies, as is often the case. Furthermore, the Hinxton Group’s recent attempt to apply their recommendations to the specific Asian regional context may have resulted in an over emphasis of cultural differences [28] which in turn did not provide concrete guidelines to ensure open access.

Several funding bodies have adopted granting policies that fulfill certain aspects of the Hinxton Group’s recommendations. Notable examples (further discussed) are: Canada’s Stem Cell Network’s IP Protection Fund and Impact Grant and the UK’s Biotechnology and Biological Sciences Research Council’s Data Sharing Plan Compliance Monitoring.

#### Summary—International Groups

International Groups involved in scientific research have been among the first and more active supporters of open access [29]. Given the mandates of ISSCR and the Hinxton Group to promote collaborative international SCR, it is no surprise that their policies seem to favor open access over commercialization, though they do also recognize the necessity to commercialize stem cell products and have included some accommodation to that effect. The rest of this paper however demonstrates that these policies have only had a moderate impact on stem cells innovation strategies at the national level.

Since more traditionally “non-profit” institutions (e.g. universities) increasingly partner with “for-profit” ones, policies need to accurately reflect this new reality. International SCR organizations must address this challenge in order for their open access policies to provide truly meaningful guidance to the global stem cell research community.

### European Union

Concerns over the moral (and *a fortiori* the legal) status of the human embryo have potentially limited the downstream commercialization of hESC-based SCR (and their clinical translation) in the European Union (EU). This is best exemplified in the landmark case of *Oliver Brüstle v Greenpeace* [30] in which the European Court of Justice ruled—based on the interpretation of the European Directive on the legal protection of biotechnological inventions (98/44/EU)—that procedures involving hESCs are unpatentable if they derive from the destruction of human embryos [31, 32]. In this decision, the Court adopted a broad definition of the term “embryo” despite the absence of consensus among the EU member states on the term, effectively replacing existing definitions of “embryo” in various national patent laws of the member states [32]. While an analysis of the patentability of SCR-based procedures and products is beyond the scope of this article, the impact of this recent EU ruling on stem cell innovation policies in the European Union should not be neglected.

#### The 7th Framework Programme (FP7) (until 2013)

The FP7 was established by the EU in 2007 for a period of 7 years. It established the notion of “European added value” or “transnational quality” as qualification criterion for funding [33]. Since inception, the FP7 has funded over 30 SCR projects organized in large-scale consortia [34]. Grant requirements address how results should be shared with consortium members, affiliates and third parties [35].

The FP7 regime allows its project participants (“participants”) to set their own guidelines and rules for sharing data, which can set discretionary limits on data sharing. Core consortium members of FP7 benefit from a patent pool; more remote actors may enjoy certain access rights and even sublicenses, if the participant consents to granting sublicenses in writing (s. II.32.5; see Figure 2) [35, 36]. The Guide to Intellectual Property Rules for FP7 Projects (“Guide”) recommends the participant allow other participants in the project access to his or her “background” information [35]. However, the definition of background specifies that “it relates only to information relevant to the project (i.e. *needed* to implement the project or *needed* to use the foreground generated)” [35] Accessing the background of another participant can only occur during the project’s duration, or within 1 year of the project’s completion [35]. Moreover, the Guide states that foreground IP (IP created during the FP7 project) “should be protected” [35] by patents, when it has industrial or commercial potential. Such arrangements for ownership and IPR can restrict access, since the participants have an opportunity to decide how “open” their research will be, unless they are receiving specific grants that have their own particular open access requirements [35].

The default IP regime by FP7 allows participants to retain exclusive rights and licensing, which encourages commercialization. Joint participants must agree among themselves to the allocation of ownership for the foreground IP (s. II.26.2) [36]. When no joint agreements exist, the FP7 Commission has a default IPR regime, where each joint owner is entitled to grant non-exclusive licenses to third parties without any right to sublicense (s. 40.2) [35–37]. As a report sponsored by the European Commission notes, exclusive licensing is a potent commercialization enabler: “[exclusivity] increases the (…) potential strength and value of their IPR and the likelihood that the results will be exploited” [38]. Therefore, the exclusive rights established by the default IPR regime can be seen as favoring commercialization.

The Commission retains the right to object to the grant of an exclusive license to parties outside the EU [35]. Regional economic considerations can therefore trump the translation of scientific research into products and services [39]. If the Commission objects to a certain project, it requests the participant to immediately suspend the granting of the license. The Commission will then communicate safeguards it considers appropriate to alleviate the objectionable grounds [35].

Finally, it should be noted that IPR protection mitigates the obligation to disseminate results to the research community at large. Under FP7, dissemination activities “shall be compatible with the protection of intellectual property rights, confidentiality obligations and the legitimate interests of the owner(s) of the foreground” (s. II.30.2) [36]. IP protection can therefore delay dissemination of research findings until a patent application is filed, as prior disclosure would invalidate the application [35]. Disproportionate harm to the IP of other project participants is also a legitimate reason to limit dissemination of the research results. The Guide to IP Rules confirms that dissemination of data is subsidiary to IPR protection: “[w]here dissemination of foreground **does not adversely affect** its protection and use, there is an obligation to disseminate it swiftly” (emphasis added) [35].

Despite these limitations on data sharing, certain initiatives under the FP7—where SCR projects may take place—do provide concrete obligations to openly disseminate research results. For example, a 2008 “Open Access Pilot” project required researchers to “deposit their articles or manuscripts in a relevant repository immediately upon acceptance for publication, to be made open access within 6 months” if the research falls under the area of Health, and within 12 months if the research falls under Science in Society [33].

#### Summary—EU

The FP7 program innovation framework reveals a restricted access model (protected commons) that only allows limited access to promote economic benefits within Europe. The grant criterion of “European added value” necessarily requires researchers within Europe to share information freely, but also constrains the ambit of that access within the parameters of the EU. Although the FP7 program appears to favor a protected commons approach, SCR grant applicants may also face obligations to disseminate research more openly from specific initiatives like the Open Access Pilot Project. However, the definition of dissemination in these open access requirements displays enough flexibility to accommodate commercialization priorities.

The various nuances and exceptions present in their policies could be challenging to navigate for scientists with limited business experience. Given the current patent conundrum, it would also be worthwhile to empirically assess how relevant these rules are for current grantees and how frequently they are being used.

## National Organizations

### United Kingdom

hESC Research in the UK is governed by the *Human Fertilization and Embryology Act* 2008 (HFEA). The administrative rules of the HFEA contained in their Code of Practice require that the cell line be deposited in the UK Stem Cell Bank (UK SCB) as a condition of the grant of a license for newly derived hESC lines, whether they are supernumerary from in vitro fertility treatments or created specifically for research purposes [40].

#### UK Stem Cell Bank

The UK SCB is the regulator mandated to be a repository for all embryonic stem cell lines derived in the UK. The SCB operates in accordance with the principles of governance laid down in its Code of Practice [41] and employs an IPRs management model tailored to the commercial potential of different types of SC lines.

The UK SCB mandates open access to laboratory-grade stem cells useful to fundamental research. While laboratory-grade lines may contain impurities limiting therapeutic applications, they are nevertheless valuable for fundamental research. The UK SCB requires sharing of these lines through a non-exclusive royalty-free license (s. 3.3) [42].

The UK SCB’s approach preserves the commercial potential of valuable clinical-grade stem cells, conducive to therapeutic in vivo applications in humans. Strict quality standards established by the European Union’s *Tissue and Cells Directives* from 2004 to 2006 (EU) govern these lines. Parties can negotiate access to clinical-grade lines for commercial purposes without restrictions from the Steering Committee (s. 6.4) [41] (ss. 1.1(m); 2.5. 2.8) [43]. This hands-off approach from the UK SCB is an incentive to commercialize, as it removes encumbrances from the IPRs. Furthermore, IPRs resulting from research by users on clinical-grade lines do not have to be licensed back to the Depositor, thereby providing private investors with a more attractive IPR portfolio.

The UK SCB’s two-track policy addresses the specific market potential of research and laboratory grade stem cells. It promotes open access to the former, while preserving the commercial value of the latter.

#### Biotechnology and Biological Sciences Research Council

The Biotechnology and Biological Sciences Research Council (BBSRC) is part of the Research Councils UK, funded by the Department for Business, Innovation and Skills. The BBSRC invested £12.3 M in SCR in 2007–08 [44]. It prioritizes the replacement of animal models with cardiomyocyte stem cell models [45]. It has also identified regenerative medicine as a priority in its Strategic Plan [44]. The Strategic Plan states that “[p]ublicly-funded research data are a public good, produced in the public interest” and “should be openly available to the maximum extent possible” [44].

The knowledge exchange and commercialization policy encourages researchers to pursue open access by stating that there are “circumstances where the publication of research outcomes or free dissemination to business might be the most effective approach” [46]. Those values are reflected in the Grants guide, which requires all applications to have a data sharing policy or justify why sharing would not be appropriate (s. 4.20) [47]. The implementation of this plan must be summarized in the final report, which counts towards the grantee’s track record for future applications [47], thus ensuring accountability.

On the other hand, the BBRSC’s IP policy encourages commercialization to some extent by identifying the securing of IPRs through funded research as a way to strengthen the UK economy. Its Policy on Maximizing Impact of Research aims to “encourage an entrepreneurial culture within the academic bioscience research base” [48]. The BBRSC’s data sharing policy allows a delay in publication if securing IPRs requires “periods of exclusive use of data” [49]. This is not pressure to commercialize per se, but rather the assurance that those interested in commercialization will have the necessary room to secure the protection of valuable innovations. The IP policy does not impose rules on the ownership of IP [50], recognizing that the varying spectrum of private sector involvement can require different patenting and licensing strategies.

Overall, the BBRSC has a strong stance on open access, as reflected in its strict requirement for grant applications to have a built-in data sharing policy. Where appropriate, it also invites researchers to commercialize via protective IPRs and provide them the necessary latitude to be successful at it.

#### Medical Research Council

The Medical Research Council (MRC) funds several research centers, including the Centre for Regenerative Medicine, which focuses on SCR. It also funds SCR projects at different stages of research through specific grants. The Royal Charter of the MRC identifies the objective of “contributing to (…) the economic competitiveness of Our United Kingdom” (s. 2(1)(b)) [51], as well as generating public awareness and communicating research outcomes, thus recognizing both open access and commercialization. Its data sharing policy states that valuable scientific data from MRC-funded research should be “made available to the scientific community with as few restrictions as possible” [52]. However, the policy also makes clear that it is “not intended to discourage filing of patent applications in advance of publication” [52] and allows a “short period” of delay in publication if necessary.

The UK-China stem cell partnership development initiative focuses on fundamental research. It seeks to develop collaborative links in stem cell research between the two countries [53]. It offers a maximum of £400,000 per collaborative project (for up to 12 projects) that aims to foster SCR at a small-scale that would eventually lead to future and joint activity. While this collaboration cannot be equated with open access because it is restricted to a finite group of scientists, it nevertheless fosters a protected environment conducive to the exchange of scientific data without the barriers of IP protection.

In addition, the Translational Stem Cell Research Committee funds research aiming to advance technological developments in preclinical SCR or early phase clinical trials. Its aim was to provide up to £10 million per year for all of its projects by 2010/11 [54]. This type of grant encourages protective IP practices in order to attract private investment. Industrial partners in translational grants can “pre-negotiate the distribution of academically generated foreground project IP” [55].

The orientation and conditions of these two SCR grants enables the MRC to offer flexible data sharing modalities. While the existence of two modes of funding allows researchers to tailor their data sharing or commercialization preference, the difference in funding amounts suggests that the MRC incentivizes commercialization more. Data sharing—restricted to research partners—may be better suited for fundamental research under the China-UK grant, while IPR strategies can secure return on investment for translational grantees working on SCR further down the developmental timeline. In sum, the MRC innovation policy seems to generally favor commercialization over open access through its general policies and funding Schemes.

#### Technology Strategy Board

The Technology Strategy Board (TSB) is the UK’s innovation agency. Its remit is to “accelerate economic growth by stimulating and supporting business-led innovation” [56]. Originally established in 2004 with an advisory role only, it became a Non Departmental Public Body with a wider remit and executive functions in 2007. It is sponsored by the Department of Business, Innovation and Skills. The TSB has funded ten SCR projects since 2010 via its Knowledge Transfer Partnerships [56] and committed over £2 million to SCR within its “Regenerative medicine—stem cells for safer medicine” program [57]. The TSB also offers £1 million in grants to study bipolar affective disorder using iPS cells [58]. Several grants encompassing SCR, including two regenerative medicine funds, seek to speed up the market-readiness of SC innovation. Grant applications must include a route to market and long-term commercialization plan [59].

The funding rules, adopted by the TSB, do not directly mandate open access. Instead, they distinguish between projects involving ‘economic activities’ in which Catapults, Public Sector Research Establishments, Research Council Institutes, charities, public sector organizations, others take part and ‘non-economic research activities’. It is envisaged that those involved in ‘economic activities’ will adopt similar practices as industrial partners in ‘normal market conditions’, including decisions *not to* disseminate results [60]. By contrast, there is an expectation that ‘non-economic activities’ will be disseminated. Dissemination through ‘open access’ repositories is one of the means envisaged. Other means extend to “[…] producing a case study, speaking at a conference, publishing academic papers, open access repositories (databases where raw research data can be accessed by anyone), or through free or open source software and so on” [60].

The TSB supports patent filing costs up to £5,000 [61], making protective IPR practices an attractive option. Researchers are thus encouraged to share their innovation in a way that is conducive to commercialization. For example, the CRACKIT grant IP scheme is designed to offer industry sponsors early access to new technology. Furthermore, access can be extended beyond the project duration “through granting of royalty-free licenses or through favorable pricing for an agreed period” [58]. The invitation to tender also requires that SC scientists ensure the availability of their work: “through publication and dissemination, or by appropriate licenses, royalty-free or royalty-bearing on fair and reasonable terms” [58]. Because sharing this material entails administrative costs and uncertainty relative to the extent of the rights conceded, researchers may be tempted to adhere to a minimal open access standard through the dissemination of publications rather than sharing the patented material or the process itself.

Finally, public funding of stem cell research in the UK is driven by a wider strategic goal to strengthen public-private partnerships and collaborations between universities and industry initiated under the Blair and Brown governments and exemplified by the UCL-Pfizer collaboration to develop pioneering stem cell sight therapies in 2009 [62]. In its 2011 report to Parliament, David Willetts, the Secretary of State for Business, Innovation and Skills announced the Coalitions’ commitment “to the UK knowledge base by maintaining the annual £4.6 billion budget for science and research programmes, with £150 million each year supporting university-business interaction which in turn benefits clusters, through Higher Education Innovation Funding” [63]. This strategy prompted the creation of the Cell Therapy Catapults established by the Technology Strategy Board in 2012 to grow the UK cell therapy industry, increasing health and wealth for all [64].

In addition, in April 2012, a report from the UK Medical Research Council (MRC) setting out “A UK Strategy for UK Regenerative Medicine” [65] signaled the intention of the leading funders (BBSRC, Engineering and Physical Sciences Research Council (EPSRC), Economic and Social Research Council (ESRC), MRC & Technology Strategy Board (TSB)) to support cell based research “through response mode funding, and continued strategic investment in centres of excellence and partnerships with industry.” A “Cell Therapy Catapult” has been launched at the end of 2013 [64, 66]. Like all the six catapults funded by the TSB, the Cell Therapy Catapult is intended “to bridge the gap between business, academia, research and government” [67].

In its 2011 report on Innovation and Research Strategy, the Coalition government announced its intention to promote open access to publications of publicly funded research alongside a commitment to strengthen support for public-private partnerships. A working group was appointed to make strategic recommendations on the implementation of open access policies. The report was endorsed by the government [68, 69].

#### Summary—UK

Public funding of SCR in the UK is taking place within a strategic framework aimed at accelerating the translation of basic research into innovative therapeutic products through close collaborations and partnerships between academic researchers and industry. The funding models and policies seek to integrate traditional modes of IP protection into public research institutions rather than displace or disrupt the commercial interests of for-profit partners through the classic divide between ‘economic’ and ‘non-economic’ research activities. Whilst the model envisages that the latter will still be undertaken by non-for profit organizations and universities who are also required to ‘disseminate’ their research, open access is currently not mandated in this environment.

### Spain

Akin to the UK approach, Spain has adopted a comprehensive framework regulating SCR and its clinical translation [70, 71]. With regards to commercialization, the *Royal Decree 55/2002 of 18 January on the Use and Assignment of Inventions in Public Research Bodies* establishes the framework for the use and assignment of inventions in public research bodies, regulating IP from public research centers such as the Spanish National Research Council (Consejo Superior de Investigaciones Cientificas, CSIC) and the Carlos III Health Institute (ISCIII), both of which are involved in SCR. The CSIC is a public institution whose primary objective is to develop and promote multidisciplinary scientific and technological research and is attached to the Ministry of Economy and Competitiveness [72]. The Carlos III Health Institute is the main research organization for biomedical research in Spain. It is also attached to the same Ministry [73]. “National Organizations” of the Royal Decree assigns IPRs to the institution, but grants the inventor a third of the benefits. If the IP is assigned to a party other than the public institution where the research took place, “the public research institution will have the right to a non-exclusive, non-transferable free exploitation licence” (s. 3). Further, “[w]hen researchers perform an invention as a result of a contract with a private or public institution, the contract must stipulate who owns IPRs” (s. 5). These broad provisions allow the necessary flexibility to structure IPRs according to the specific needs of each public-private partnership and provide a predictable IPR distribution framework.

Some federal laws appear to pressure researchers to commercialize. The 11/1986 *Law on Patents* [74] requires university researchers to report patentable inventions. The 14/2011 *Law on Science, Technology and Innovation* [75] sets out a knowledge transfer policy for public R&D funders. Pursuant to this law, the “[v]alorisation of the knowledge obtained through research applies to all processes allowing publicly-financed research to reach all sectors of society. Its objectives are (…) to facilitate an adequate protection of the research results with a view to transferring such results” (s. 35(2)(b)) [75]. The law also states that the transfer of research results to third parties (via patent or licenses) are governed by contract law, where each party can establish its own protective measures (s.36) [75]. These laws set the normative background to which institutional funding policies containing indications of substantial pressures to commercialize academic SCR are developed.

#### Spanish National Stem Cell Bank (SNSCB)

The Carlos III Health Institute is responsible for the Spanish National Stem Cell Bank (SNSCB), a decentralized network of hESC and iPSC repositories ensuring nation-wide access for non-commercial research. It combines intervention to ensure access to basic science material with a hands-off approach to commercial transactions. Public and private institutions deriving SC lines must deposit a sample in the SNSCB (*Order SCO/393/20*). Licenses governing access to these lines are available only to researchers operating in non-profit settings (s. 3) [76]. This excludes any private involvement: “material shall not be used in a research program where rights (either actual or contingent) have already been granted to a research sponsor who does not have a separate written agreement with provider permitting such use of material” (s. 3) [76]. However, the bank refrains from intervening in negotiations with potential commercial buyers.

#### The autonomous community of Andalusia

The community of Andalusia provides a distinct setting, within the Spanish national jurisdiction, for SCR to develop. The *Law 16/2007 on Science and Knowledge* governs the management of science in Andalusia [77]. Its provisions emphasize commercial potential (s. 53 (a)–(b)), and it sets out incentives such that: “[a]chieving an invention susceptible of protected exploitation through intellectual property rights (…) shall be meritorious in the evaluation criteria of calls, employee selection and professional development in the Andalusian public sector” (s. 60). Researchers can receive a percentage of the royalties their inventions earn for public entities.

The Andalusian Public Health System’s Technology Transfer Office (TTO-APHS) has MTAs applicable to the sharing of SCR. It proposes two agreements for the exchange of research material between public institutions. The first model of MTA postpones determination of IP ownership of subsequent discoveries: “[i]f research results related to the material are susceptible of legal protection, the parties shall meet with their legal representatives to determine invention authorship and the contribution of each party” (s. 7) [78]. This delay in determining ownership creates uncertainty over who owns the rights to inventions arising from shared cell lines. Researchers may be more reluctant to collaborate under this MTA if they wish to preemptively secure IP ownership over their future inventions.

The TTO-APHS is a signatory to the *UBMTA* of the Association of University Technology Managers [79], dealing with the transfer of unpatented material between non-profit entities. Users deriving a patentable process or product own the rights to their inventions and can share them for non-commercial purposes (s. 8) [79]. This research can be commercialized if the derived product is sufficiently different from the original material (s. 5(a)) [79]. Otherwise, the commercialization of inventions that incorporate the original material is subject to negotiations with the provider of the material (s. 7) [79]. The *UBMTA* allows providers and users of SC material more certainty about the ownership and use of subsequent innovations, and could significantly reduce barriers to open access sharing of non-commercial research. The fact that the *UBMTA* is a well recognized international standard should also facilitate negotiations and potentially speed up the transfer process.

#### Andalusian Initiative for Advanced Therapies (AIAT)

The AIAT is funded by the government of the Autonomous Community of Andalusia’s government, through its Ministry of Health and the Regional Ministry of Economy [80]. This public agency seeks to translate SCR into therapies without private involvement. Instead of relying on private commercialization to achieve translation, the AIAT extends state support to later-stage research. It sponsors clinical trials driven by public local institutions and hospitals. In doing so, it aims to translate discoveries that have therapeutic potential but few commercial prospects.

#### Summary—Spain

Federal laws in Spain encourage commercialization by providing the flexibility necessary to accommodate the needs of those who wish to take the commercialization route. Similar to several other surveyed jurisdictions, Spain’s approach incentivizes research with commercial potential by providing public researchers with a margin of the benefits from IPR exploitation. Regional laws in Andalusia reveal a similar stance. Broad legislative statements about knowledge transfer through IPR protection are made at both levels of government and some limited initiatives exist at the regional/institutional levels to support open access and non-commercial research.

### Canada

hESC research is permitted in Canada, as long as it takes place within a specified ethical and legal framework as circumscribed by a patchwork of federal and provincial legislation, in compliance with a Supreme Court ruling that challenged the constitutionality of the federal Assisted Human Reproduction Act [81]. SCR is also subject to various research guidelines [82, 83].

#### Canadian Institutes of Health Research (CIHR)

CIHR has a mandate to foster collaboration and facilitate commercialization [84]. In 2012, the CIHR, in partnership with Genome Canada, assigned $67.5 million for the Genomics and Personalized Health program [85], and $10 million for a Program Grant in Transplantation Research [86] and $6 million for Canada-Japan Teams in Epigenetics of Stem Cells [87].

CIHR’s grant guidelines contain broad statements and a few non-specific requirements on open access [88] or commercialization. According to the updated *2010–2012 Guidelines for Human Pluripotent Stem Cell Research*, all hESCs derived with CIHR funding must be “listed with the registry [established by CIHR] and made available by the researcher to other researchers” (s. 6) [89]. Grants require a knowledge dissemination and exchange strategy [90] and evaluation criteria include “[a]ppropriateness of the proposed strategies for Knowledge Translation” [91]. The grants awards guide further states that “[t]he research process is not complete until the results are validated and openly transmitted to the appropriate audience” (s. 2-A21.2) [92].

Commercialization incentives are equally sparse and indirect in the CIHR guidelines, which do not provide any guidance or facilitation on IPRs. The Tri-Agency policy, applicable to the three major federal life science funders including the CIHR, delegates IP management to the researcher’s institution [93]. The hands-off approach regarding commercialization is also apparent in its statement: “[t]he Agencies (…) do not pass judgment on the eventual commercial success of the research” [93]. Despite this attitude towards commercialization, certain CIHR grant applications seem to put an emphasis on commercialization potential and plans as criteria for funding [94].

#### Stem Cell Network

The Stem Cell Network (SCN) is a Networks Centre of Excellence, whose activities consist of R&D and “translation-commercialization” [95]. Organized in “large-scale, academically-led virtual research networks that bring together partners from academia, industry, government and not-for-profit organizations” [96], it recently decided to fund SCR through the “valley of death” stage, i.e. “too late for traditional academic grants but too early for industry sponsorship” [97]. Its budget amounts to over $82 million for the 2001–2015 period [95].

The SCN prioritizes commercialization by providing an optional IPR management model and separate grants for market translation. These grants do not necessarily conflict with open access because the aforementioned strategies are optional and separate from open access requirements. It also offers incentives to commercialize SCR with market potential, while remaining alert to the importance of open access to fundamental research. Although the SCN’s goal is to “accelerate projects with commercial and therapeutic potential” [98], this pull is achieved through soft incentives. Its IP Protection Fund [99] and Commercialization Impact Grants [100] offer $5,000 and $75,000 respectively to initiate and refine IP assets developed with SCN funding. In the core grants, the researcher must detail a long-term clinical translation plan, but this can take the form of a protective IPR strategy or open-access data sharing. Scientists are simply asked to “[p]rovide a brief and clear description of [their] long-term clinical translational plan for the proposed research including how the proposed grant will further the long-term goals” [101]. The message sent to the researcher is that the SCN will use additional funding to promote commercialization as the best route to translation, but open access to the research remains an option. Thus, the SCN exerts a soft pressure on its researchers to commercialize via additional strategic grant opportunities, training seminars [102] and the disseminations of commercialization tools [103] without proscribing open access.

The SCN provides a set of boilerplate IP agreements [103] to assist academic and industrial partners wishing to collaborate on commercializing SCR. By providing a comprehensive IPR framework, this toolkit could save significantly on transaction costs associated with IP management. The toolkit also pre-empts possible IPR ownership conflicts, thus reducing costs associated with potential litigation. Echoing the UK SCB’s provisions for downstream inventions, these agreements give privileged access to scientists who derive a stem cell [104].

SCN’s attempt in 2005 to promote the commercialization of stem cell research via the creation of a spinoff company, Aggregate Therapeutics, was unsuccessful. Aggregate Therapeutics was incorporated with the goal of managing and leveraging the intellectual property of interested researchers from the SCN. However, the company did not gain enough private sector investment due to a host of reasons, including the early stage of research and uncertainty about IPRs [3]. The early-stage nature of IPRs owned by Aggregate Therapeutics also did not fit with the fundraising model of venture capital investors who required shorter timeframes than the IP allowed [105]. Interestingly, Aggregate Therapeutics kept a clause in its model licensing agreement that allowed SCN researchers to be able to use the technology for non-commercial purposes [106]. After the demise of Aggregate Therapeutics, the drive for commercialization of SCR has continued with the emergence of the Centre for Commercialization of Regenerative Medicine (CCRM) in 2011. The Centre explicitly aims to “accelerate the commercialization of stem cell- and biomaterials-based technologies and therapies” [107].

#### Summary—Canada

In the context of SCR, CIHR favors a hands-off approach to commercialization as illustrated by the broad requirements for open access in its policies and the absence of commercialization requirements or guidelines. The CIHR Program in Transplantation Research, a notable exception to this approach, reveals a certain preference towards commercialization by inviting grant applicants to identify previous patents or IPRs. The SCN exerts soft pressure on its researchers to commercialize their work through additional funding opportunities and its extensive commercialization/IP reference documents. Yet ultimately, there is no conflict between open access and commercialization requirements, since these components are optional.

### United States

In contrast with the other jurisdictions under study, the United States does not have a comprehensive legislative scheme addressing stem cell research. Instead, it controls hESC research through restrictions on federal science funding. The 1996 Dickey-Wicker amendment limited Congress’ ability to fund embryo research, curtailing HESC line derivation with federal funding [108]. From 2001 to 2009, President Bush’s *Executive Order 13435* (2007) limited federal funding for stem cell research by prohibiting research using HESC lines derived after 2001. However, President Obama lifted this restriction with *Executive Order 13505* in 2009, which was later implemented via the *National Institutes of Health Guidelines for Human Stem Cell Research* [109]. Interestingly, the above restrictions are not applicable to privately funded hESC research.

The *Bayh-Dole Act* (1980) [110] amended federal patent law by changing the allocation of intellectual property for federally funded research. It encourages commercialization by allowing non-profit organizations (including universities) to retain ownership of their inventions when they conduct research with federal funding. These organizations are expected to prioritize commercialization and give preference to small US-based businesses. Any open access requirements by funding bodies must be compatible with the *Act*.

#### National Institutes of Health

The NIH invested over $1 billion in stem cell-related research in 2011 [111]. It conducts research through in-house institutes such as the Center of Regenerative Medicine, and outsources funding to universities and research institutions. 80 % of its budget goes towards outsourced research for which the NIH only acts as a funder [112]. It imposes dual obligations on researchers; they must disseminate unique research resources to develop science, and commercialize their inventions to demonstrate the economic benefits to taxpayers [113].

The Policy on Sharing of Model Organisms for Biomedical Research [114] lays out open access modalities for NIH-funded research in a model MTA. Noting that the “NIH is able to support more investigators than if these useful models had to be generated in duplicate by more than one NIH funded investigator” [114]. It encourages free dissemination of unpatented research materials such as subclones of unmodified cell lines and genetically modified organisms among NIH-funded researchers.

The *Principles and Guidelines for Recipients of NIH Research Grants and Contracts on Obtaining and Disseminating Biomedical Research Resources* [113] present various access policies, depending on two factors. The first concerns the position of the material along the development timeline: whether it is a research tool or a product with commercial potential. The second factor is the identity of the accessing party: whether they are an NIH-funded colleague, an external non-profit researcher or a for-profit entity.

For fundamental research, scientists receiving NIH funding can access material through a Simple Letter of Agreement (SLA). External non-profit researchers benefit from a royalty-free non-sublicenseable research license governed by the UBMTA, which details how an accessing researcher can share modifications to the provider’s unpatented material. Commercial users must obtain a non-exclusive license. Access is gradually restricted as the product moves through development, from a quasi-informal SLA to a binding license. Technology closer to commercial application, particularly that in need of private investment to move forward, can be shared with a free or paying royalty scheme, depending on whether access is sought for academic purposes of conducting further research or profit purposes of commercialization. According to the *Principles and Guidelines for Recipients of NIH Research Grants and Contracts on Obtaining and Disseminating Biomedical Research Resources*, even exclusive licenses are acceptable [113]. Under this model, stem cell researchers would be required to share their research tools and products if their research is related to public health.

More recently, the NIH’s *Best Practices for the Licensing of Genomic Inventions* recommended “licensing policies and strategies that maximize access, as well as commercial and research utilization of the technology to benefit the public health” [115]. These guidelines are likely relevant for SCR, as they apply to “full-length genes and their expression products” [115].

Each grant project seeking over $500,000 per year must submit a data-sharing plan. *The Data Sharing Policy and Implementation Guidance* [116] suggests several methods of sharing: researchers can manage access requests themselves, or deposit data in an archive or enclave. If researchers do not plan to share the data, they must justify their refusal. In cases of non-compliance, the NIH may “make data sharing an explicit term and condition of subsequent awards” [116]. However, the Policy does not stipulate specific content, formatting, modes, or timeframes for data sharing and simply states: “what is sensible in one field…may not work at all for others” [116]. Moreover, within the NIH there are numerous data sharing policies stipulating different requirements for data sharing from its subdivisions such as National Institute of Allergy and Infectious Diseases (NIAID) [117]. Some of these data sharing policies stipulate that research data must be available within one year of publication [118] while others sharing plans are more open-ended.

In sum, the NIH recognizes the importance of open access by facilitating a protected commons model for sharing research between NIH-funded researchers, and by mandating a data-sharing plan for substantial science research funding.

#### National Stem Cell Bank at the WiCell Research Institute (until 2011)

From 2005 to 2011, the NIH funded the National Stem Cell Bank (NSCB) at the WiCell Research Institute in Wisconsin. While the NSCB ceased is operations in 2011 due to the end of its agreement with the NIH, it is important to analyze it as it provides an illustration of a protected commons approach used to alleviate the tension between open access and commercialization requirements. Because researchers procuring SC lines from WiCell for NIH-funded research were granted a non-commercial license, it imposed several access obligations on derived material, inventions or products the users created, thereby creating an arrangement that can be qualified as a limited sharing model.

The Memorandum of Understanding between the federal government and WiCell precluded users from commercializing hESC lines from WiCell and the patents belonging to the Wisconsin Alumni Research Foundation, but allowed the commercialization of materials or processes derived from the user’s work. Users were able to commercialize their own work, but the IP was subject to a non-commercial research license benefiting the NSBC, the provider of the line, and certain non-profit and academic institutions [119–121]. In both cases, non-commercial licenses to users’ work on the stem cell lines were considered a *quid pro quo* for allowing initial access to the material.

#### California Institute of Regenerative Medicine (CIRM)

The CIRM was created as a result of Proposition 71 (also known as the *California Stem Cell Research and Cures Act*) [122]. Similar to Canada’s SCN, CIRM’s objective is to fund projects in the “Valley of Death” stage, where research in early stages does not have enough federal funding to reach the clinical trial stage that is often funded by private industries [123, 124].

As the goal of Proposition 71 was to benefit Californians with “rapid advancement” of stem cell research (s. 2) [122], CIRM prioritizes access to materials within the state. In this sense, its sharing policy is akin to a protected commons limited to California. CIRM-funded researchers must share newly derived lines with other researchers after publication, as per the *California Code of Regulations*: “[a] Grantee shall share Publication-related Biomedical Material, for bona fide purposes of research in California” [125]. Since its definition of research encompasses commercial activities like “research development, testing and evaluation, designed to develop or contribute to generalizable knowledge” (s. 100020 (i)) [125], California’s private SCR companies have access to SC lines related to CIRM-funded publications before foreign non-profit SCR institutions.

The *raison d’être* of Proposition 71 was to make medical treatments and cures more available for uninsured California residents (s. 100607) [125]. The language of the regulations and requirements provide a flexible framework where open access principles are dynamically balanced with commercialization imperatives. However, the regime does advantage California residents over foreigners.

The CIRM’s IPR policies are embedded in the health regulations of the *California Code of Regulations*, ensuring clout and uniformity. There is a general obligation to “make reasonable efforts to develop, commercialize or otherwise bring to practical application CIRM-Funded Technology or CIRM-Funded Inventions” ( s. 100606(a)) [125]. A third party can implement this requirement by licensing the patented technology (s. 100606(b)) [125], but the CIRM-funded researchers remain responsible for ensuring that their technology become publicly available. In the case of exclusive licensing, CIRM retains reach-through rights to appoint other licensees to carry the research forward (s. 100610) [125].

CIRM’s IPR regime also sets out a series of rewards to the State depending on the volume of licensing revenue and the value of the CIRM’s initial investment, creating a quasi joint venture between the State and the grantee. (s. 100608 (a)(2); (b)(1)) [125]. These policies appear to have resulted in economic growth in California, as illustrated in one study that found a 30 % increase in venture capital investments of life sciences from the 2006–2011 period (the first 5 years of CIRM) as compared to the period between 2000 and 2005 [16].

#### Summary—United States

An examination of US policies reveals that federal public funding bodies most commonly mandate limited access to materials and data (i.e. protected commons limited to state or national borders). This may be due to the fact that much of the commercialization pressures come from federal or state laws. Certain organizations, like the CIRM, have developed their own regulations into a framework where strong research commercialization requirements do not seem to jeopardize accessibility due to a sharing model similar to a protected commons. In addition, the move towards a greater focus on stem cell research and regenerative medicine in California will certainly have some impact on the future of geographic distribution of universities, biotechnology and pharmaceutical infrastructures and investments of venture capital in the United States [124].

Because philanthropic organizations also contribute significantly to SCR and its clinical translation in the U.S., their funding requirements will also shape commercialization and open access pressures. This is an area where further research is needed. In sum, in the US the pressure to commercialize arguably remains strong because commercialization requirements are embedded in federal legislation and certain state regulations.

## Discussion

The purpose of our research was to investigate and lay out access and commercialization pressures that exist in public funding policies and documents from SCR organizations. We also investigated the level of integration between the various requirements contained in policies governing embryonic SCR.

Except for the international level which clearly favored openness, the policy framework of all surveyed jurisdictions proved flexible enough to permit both open access and commercial approaches to technology translation in the field of stem cell research. While some countries, such as the United States, seemed to exert stronger pressure towards commercialization; the actual differences were just a matter of degree, rather than a case of completely distinct or opposing policy positioning. The latter, coupled with the fact that the studied frameworks were formulated in broad policy strokes (as opposed to extensive legal regulations), renders impossible a micro level comparative assessment of the various national approaches. However, we can still glean some interesting trends emerging when looking at the broad lens of our research objective, this is, to compare and contrast the pressures of both open access data sharing and commercialization in public funding policies and stem cell innovation frameworks.

Our survey of policies by public funding bodies reveals subtle pressures to commercialize research that include increased funding availability for commercialization opportunities, assistance for obtaining IPRs and federal legislation directly mandating commercialization. A substantial amount of the pressure to commercialize could also originate from normative documents generally applicable to public research, not directly related to SCR. Although international groups like the ISSCR and the Hinxton Group have been advocating open access models, their limited political sway and the lack of associated financial incentive limit the impact of their recommendations.

Another thread emerging from our analysis is the appearance of a protected commons model (i.e. access limited to researchers residing within the country) as a compromise between open and proprietary SCR. Some institutions grant priority access to a limited set of actors based on a common funder (NIH) or jurisdictional belonging (CIRM). Others restrict access through bilateral funding arrangements such as the UK-China stem cell partnership development initiative at the MRC. This protected commons model could be considered a compromise when innovations are too advanced to be shared openly like basic research, but not sufficiently mature to sustain a proprietary model designed for market-ready products. Nevertheless, we also see it applied to basic research (NIH, MRC UK-China partnership), suggesting a withdrawal from the completely open access innovation model. These more restrictive IPR practices must be situated within the policy framework governing hESC in each country, since some regions such as the EU forbid patenting procedures involving human embryonic stem cells. It should be clearly understood that these controlled access national schemes do not equate to open sharing. Sooner or later, the international research community will need to position itself on the necessity of these more protectionist requirements.

Our research also sheds substantial light on the technology transfer “grey zone” of policies, identifying a protected commons approach between open access and commercialization that is emerging in SCR. This grey zone is further complicated by the evolution of universities from being “non-profit organizations” to also playing a significant entrepreneurial role [126].

Potential confusion might arise because general data sharing policy for health research or national innovation frameworks may differ from specific requirements in grants pertaining to SCR. For example, the innovation framework of the FP7 funding body strongly prefers commercialization and allows researchers to delay publication if necessary, yet their Open Access Pilot Project requires immediate deposit of research findings in a public repository.

There is also a need for more standardized definitions of terms such as “academic research” (sometimes assimilated with “not for profit research”) and “commercial purposes” (sometimes assimilated with “for profit research”) to alleviate confusion and uncertainty for researchers. With increasing partnerships between universities and for-profit organizations to foster commercialization, universities are straddling the boundary of not-for-profit research and commercialization [127]. It is not always clear how funding bodies distinguish a non-profit researcher from for-profit researchers, who would be subject to different requirements for sharing modifications to a provider’s material.

In conclusion, stem cell researchers are usually guided through various push and pull mechanisms to make one of the three choices: 1) open their research to the international community; 2) deposit their research in a protected common that will be made accessible for a specific class of research or to a specific type or researchers; 3) commercialize their research through intellectual property, licensing and public private partnerships. Preferred strategies and outcomes, as well as the degree of complexity of this stem cell innovation maze vary greatly according to each investigated jurisdiction. Integrating these policies to promote international collaborative stem cell research will no doubt be a daunting task. Yet, given the current difficulties translating stem cell science from bench to bedside, the SCR community should move towards aligning policies, in order to prevent further negative impacts to an already complex process.

## Abbreviations

hESC: human embryonic stem cells
iPSC: induced pluripotent stem cells
IPRs: intellectual property rights
MTA: material transfer agreement
SCR: stem cell research
SLA: simple letter agreement
TTO: technology transfer office
